# Colocalization and Interaction Study of Neuronal JNK3, JIP1, and β-Arrestin2 Together with PSD95

**DOI:** 10.3390/ijms23084113

**Published:** 2022-04-08

**Authors:** Clara Alice Musi, Giacomo Marchini, Arianna Giani, Giovanni Tomaselli, Erica Cecilia Priori, Luca Colnaghi, Tiziana Borsello

**Affiliations:** 1 Department of Pharmacological and Biomolecular Sciences, Università degli Studi di Milano, Via Balzaretti, 9, 20133 Milan, Italy; clara.musi@unimi.it (C.A.M.); giovanni.tomaselli@unimi.it (G.T.); erica.priori@unimi.it (E.C.P.); 2 Mario Negri Insitute for Pharmacolgical Research–IRCCS, Via Mario Negri, 2, 20156 Milan, Italy; giacomo.marchini@marionegri.it (G.M.); arianna.giani@marionegri.it (A.G.); 3 Division of Neuroscience, IRCCS San Raffaele Scientific Institute, Via Olgettina, 58, 20132 Milan, Italy; colnaghi.luca@hsr.it; 4 School of Medicine, Vita Salute San Raffaele University, Via Olgettina, 58, 20132 Milan, Italy

**Keywords:** synaptic dysfunction, protein-protein interaction, brain diseases, neuroprotection

## Abstract

c-Jun N-terminal kinases (JNKs) are stress-activated serine/threonine protein kinases belonging to the mitogen-activated protein kinase (MAPK) family. Among them, JNK3 is selectively expressed in the central nervous system, cardiac smooth muscle, and testis. In addition, it is the most responsive JNK isoform to stress stimuli in the brain, and it is involved in synaptic dysfunction, an essential step in neurodegenerative processes. JNK3 pathway is organized in a cascade of amplification in which signal transduction occurs by stepwise, highly controlled phosphorylation. Since different MAPKs share common upstream activators, pathway specificity is guaranteed by scaffold proteins such as JIP1 and β-arrestin2. To better elucidate the physiological mechanisms regulating JNK3 in neurons, and how these interactions may be involved in synaptic (dys)function, we used (i) super-resolution microscopy to demonstrate the colocalization among JNK3–PSD95–JIP1 and JNK3–PSD95–β-arrestin2 in cultured hippocampal neurons, and (ii) co-immunoprecipitation techniques to show that the two scaffold proteins and JNK3 can be found interacting together with PSD95. The protein-protein interactions that govern the formation of these two complexes, JNK3–PSD95–JIP1 and JNK3–PSD95–β-arrestin2, may be used as targets to interfere with their downstream synaptic events.

## 1. Introduction

Mitogen-activated protein kinases (MAPKs) are a family of serine/threonine (ser/thr) kinases activated by extra- and intracellular stimuli. The canonical transduction pathway of MAPKs involves three different kinases that sequentially phosphorylate and activate each other, forming a cascade that amplifies the signal [1]. The first members of this signaling module are MAPK kinase kinases (MAPKKKs) [2]. Once activated, they phosphorylate a downstream MAPK kinase (MAPKK), which in turn activates a MAPK by dual phosphorylation on both ser/thr and tyrosine residues. Finally, MAPKs act on their substrates by phosphorylating them on serine or threonine residues adjacent to a proline [3].

MAPKs are ubiquitously expressed and regulate physiological and pathological cellular processes, ranging from gene expression to cell cycle regulation, from cell differentiation to survival and apoptosis. The localization of MAPKs within the cell determines their function. For instance, in highly polarized cells, such as neurons, MAPKs assume different roles depending on their subcellular localization, i.e., soma, dendritic spines, or axons [4]. The localization inside the cell is not the only functional regulator of MAPKs. Scaffold proteins further modulate the complexity of the MAPK signaling pathways. Scaffolds are adapter proteins that, creating a modular complex, interact in a highly dynamic manner with all the three kinases of the MAPK pathways and their substrates [5].

MAPKs are subdivided into three major groups: extracellular signal-regulated kinase (ERK); mitogen-activated protein kinase p38, and c-Jun NH2-terminal kinase (JNK) [6]. JNKs include three different isoforms (JNK1, JNK2, and JNK3), encoded by three separate genes, in humans, on chromosomes 4, 5, and 10, respectively [7]. While JNK1 and JNK2 are ubiquitously expressed in all tissues, JNK3 is mainly expressed in the central nervous system (CNS) and, to a lesser extent, in the heart, testis, and pancreas [8,9]. In neurons, JNKs target several phosphoproteins in different cellular compartments. JNKs phosphorylate their elective target, c-JUN and ATF2, in the nucleus, thus influencing gene expression. In the mitochondria, JNKs phosphorylate proteins of the BCL2 family. At the synapse, JNKs target several postsynaptic density (PSD) scaffold proteins (i.e., PSD95). Among the three JNK isoforms, JNK3 is the most responsive to stress stimuli in the brain, and it plays a crucial role in synaptic dysfunction; a condition underlying different brain diseases, such as Alzheimer’s disease, Parkinson’s disease, and stroke [4,10]. For these reasons, JNK3 is an attractive target for the development of new drugs for CNS pathologies.

Similar to other MAPKs, JNK3 is regulated by scaffold proteins [11]. To date, the two most studied JNK scaffolds are JIP1 and β-arrestin2; however, their specific interaction with JNK3 in brain cells has been largely overlooked.

JIP1 interacts with all JNK isoforms and other members of the JNK signaling cascade (i.e., MKK7, phosphatases, MEKK3, MLK3, and DLK) [12]. Still, it does not interact with, and thus does not regulate, the ERK and p38 signaling pathways [13,14]. JIP1 mRNA can be found in the cortex, hypothalamus, cerebellum, medulla, pituitary gland, olfactory bulb, and hippocampus. In the cortex and cerebellum, JIP1 localizes in growth cones in the developing nervous tissue, and in synaptic complexes in the fully developed brain [15,16,17,18,19]. Accordingly, biochemical fractionation of neuronal cells shows that JIP1 can be found in the soma and synaptic membrane fractions [15,20,21]. The interaction between JNKs and JIP1 is mediated by 20 amino acids [22,23,24] of the JNK-binding domain (JBD) of JIP1 [25,26]. The overexpression of the JBD domain or of JIP1 full-length reduces JNK pathway activation [27,28], modulating synaptic plasticity [23,29,30,31].

β-arrestin2 plays a crucial role in G protein-coupled receptor (GPCR) signaling [32], and can also act as a scaffold protein for kinases and phosphatases involved in cell signaling [33]. β-arrestin2 localizes throughout the cell body, nucleus, and also in the cilia, in cultured hippocampal neurons [34]. β-arrestin2 binds all JNKs [35]; the specificity of the interaction between JNK3 and β-arrestin2 is mediated by the unique N-terminal region of JNK3, absent in other JNK isoforms, that binds to the C-terminus of β-arrestin-2. Mutations of residues in these regions compromise the β-arrestin2-facilitated JNK3 activation [36]. Furthermore, β-arrestin2 can also influence JNK3 localization. Under control conditions, JNK3 shows a nuclear and cytoplasmic distribution; however, in Saos2 cells, it was reported that the co-expression of β-arrestin2 and JNK3 forces JNK3 outside the nucleus [37,38].

While in vitro conditions and in transfected cell lines the interaction between JNK3 and its scaffold proteins has been described in fine details, much less is known in terms of the physiological localization of these interactions in neurons.

With this aim, taking advantage of super-resolution microscopy and biochemical techniques, we analyzed JNK3 interaction with its scaffold proteins in primary neurons and whole-brain homogenates.

## 2. Results

### 2.1. JNK3 Colocalizes with PSD95/JIP1 in Brain Cells

**Figure 1:**
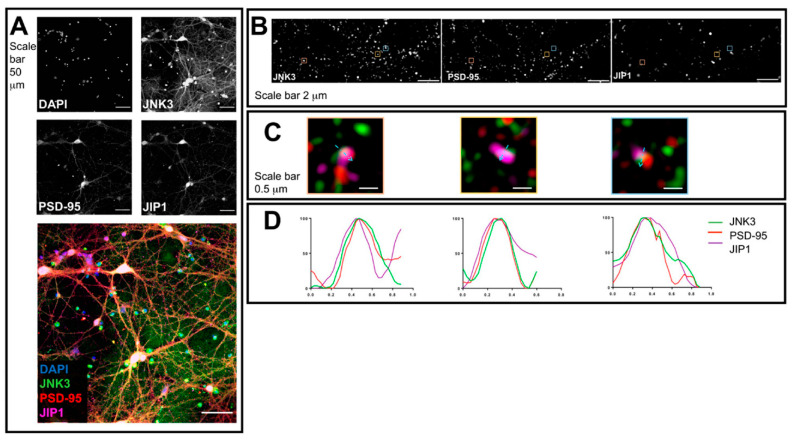
Confocal microscopy and SIM outputs used to assess JNK3–JIP1–PSD95 colocalization. (**A**) Neurons were fixed and stained with anti-JNK3 (green), anti-PSD95 (red), and anti-JIP1 (violet). Images were obtained using a Nikon N-SIM confocal microscope and overlaid to assess protein localization. Nuclei were stained with Hoechst. Scale bar of 50 μm. (**B**) 3D-SIM images were acquired with a 100× objective. Scale bar of 2 μm. Squares represent the location of the inset present in panel (**B**). (**C**) Merged images of reconstructed 3D-SIM images of JNK3 (green), PSD95 (red), and JIP1 (violet). Scale bar of 0.5 μm. (**D**) Intensity profile (green for JNK3, red for PSD95, violet for JIP1) representing the values indicated by the arrows in panel (**C**). The values are normalized to 100 (arbitrary unit).

To determine JNK3–JIP1–PSD95 colocalization, we cultured primary hippocampal neurons. At DIV14, upon fixation, we stained them with specific antibodies against JNK3, JIP1, and PSD95. Cultures were first analyzed using confocal microscopy to visualize neuronal morphology and staining, confirming a mainly cytoplasmic localization for all three proteins: JNK3, JIP1, and PSD95 (Figure 1A). Next, we analyzed neuronal processes by performing 3D structured illumination microscopy (3D-SIM). We used 3D-SIM as it is a super-resolution microscopy technique that improves the resolution of optical microscopes from 250 nm to about 125 nm, and can be used to study the subcellular colocalization of proteins [39,40,41,42,43]. We took images using a 100× objective (Figure 1B) and identified regions of signal overlap corresponding to the different proteins (Figure 1C). We next analyzed such areas by performing profile analysis of single loci, and we found that JNK3–JIP1–PSD95 can colocalize in primary hippocampal neurons (Figure 1D), thus suggesting that these proteins can form complexes in neuronal processes.

### 2.2. JNK3 Colocalizes and Forms a Complex with PSD95/β-Arrestin2 in the Brain Cells

**Figure 2:**
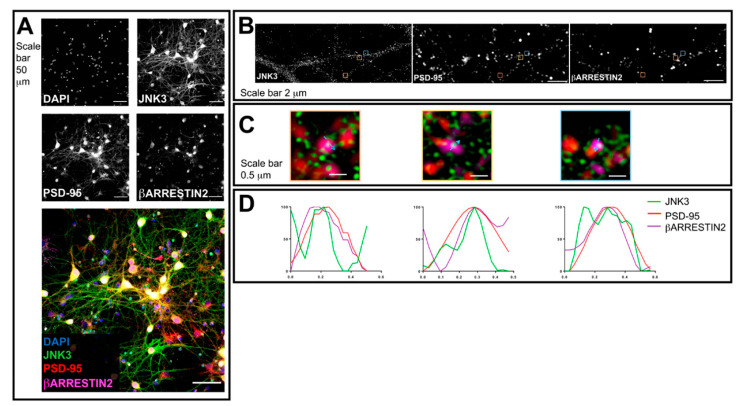
Confocal microscopy and SIM outputs used to assess JNK3–β-arrestin2–PSD95 colocalization. (**A**) Neurons were fixed and stained with anti-JNK3 (green), anti-PSD95 (red), and anti-β-arrestin2 (violet). Images were obtained using a Nikon N-SIM confocal microscope and overlaid to assess protein localization. Nuclei were stained with Hoechst. Scale bar of 50 μm. (**B**) 3D-SIM images were acquired with a 100× objective. Scale bar of 2 μm. Squares represent the location of the inset present in panel (**B**). (**C**) Merged images of reconstructed 3D-SIM images of JNK3 (green), PSD95 (red), and β-arrestin2 (violet). Scale bar of 0.5 μm. (**D**) The intensity profile (green for JNK3, red for PSD95, violet for β-arrestin2) represents the values indicated by the arrows in panel (**C**). The values are normalized to 100 (arbitrary unit).

Next, we performed a similar analysis for β-arrestin2, together with JNK3 and PSD95. We stained hippocampal neuronal cultures with antibodies against JNK3, β-arrestin2, and PSD95. The confocal images acquired with a 40× objective reveal that the staining of all three proteins shows a cytoplasmic localization (Figure 2A). Next, we switched to 3D-SIM and compared the localization of JNK3, β-arrestin2, and PSD95 in neuronal processes (Figure 2B). Profile analysis shows that JNK3–β-arrestin2–PSD95 can colocalize in primary hippocampal neurons (Figure 2C,D), suggesting that these proteins, and JNK3–JIP1–PSD95, form complexes in neuronal processes.

### 2.3. JNK3 Forms Complexes with PSD95, β-Arrestin2, and JIP1 in Hippocampal Primary Neurons

**Figure 3:**
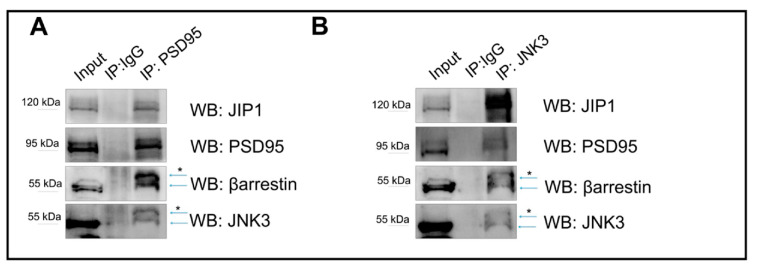
Immunoprecipitation outputs used to assess JNK3–JIP1–β-arrestin2–PSD95 colocalization in primary hippocampal neurons. (**A**) PSD95 was immunoprecipitated from the cell lysate homogenate using PSD95 antibody, and immune complexes were analyzed for the presence of JNK3, JIP1, and β-arrestin2. Immunoprecipitation with IgG antibody was used as a control. (**B**) JNK3 was immunoprecipitated from the cell lysate homogenate using JNK3-specific antibody, separated by SDS-PAGE, and analyzed via Western blot with anti-PSD95, anti-JIP1, and anti-β-arrestin2 antibodies. Immunoprecipitation with IgG antibody was used as a control. * indicates non-specific bands.

To determine whether JNK3, JIP1, β-arrestin2, and PSD95 interact, we performed co-immunoprecipitation assays starting from protein extracts obtained from cultured murine hippocampal primary neurons. First, we immunoprecipitated the protein PSD95 (IP-PSD95). The PSD95 pull down immunoprecipitated JNK3, β-arrestin2, JIP1, and PSD95 (Figure 3A). Second, we performed a similar experiment, but this time we pulled down JNK3 (IP-JNK3) and tested for interactions by Western blotting. We confirmed that all proteins can be co-immunoprecipitated (Figure 3B).

Overall, together with the super-resolution evidence (Figure 1 and Figure 2), the data suggest that JNK3 colocalizes and interacts with β-arrestin2, JIP1, and PSD95.

### 2.4. Localization of JNK3 in Primary Hippocampal Neurons

**Figure 4:**
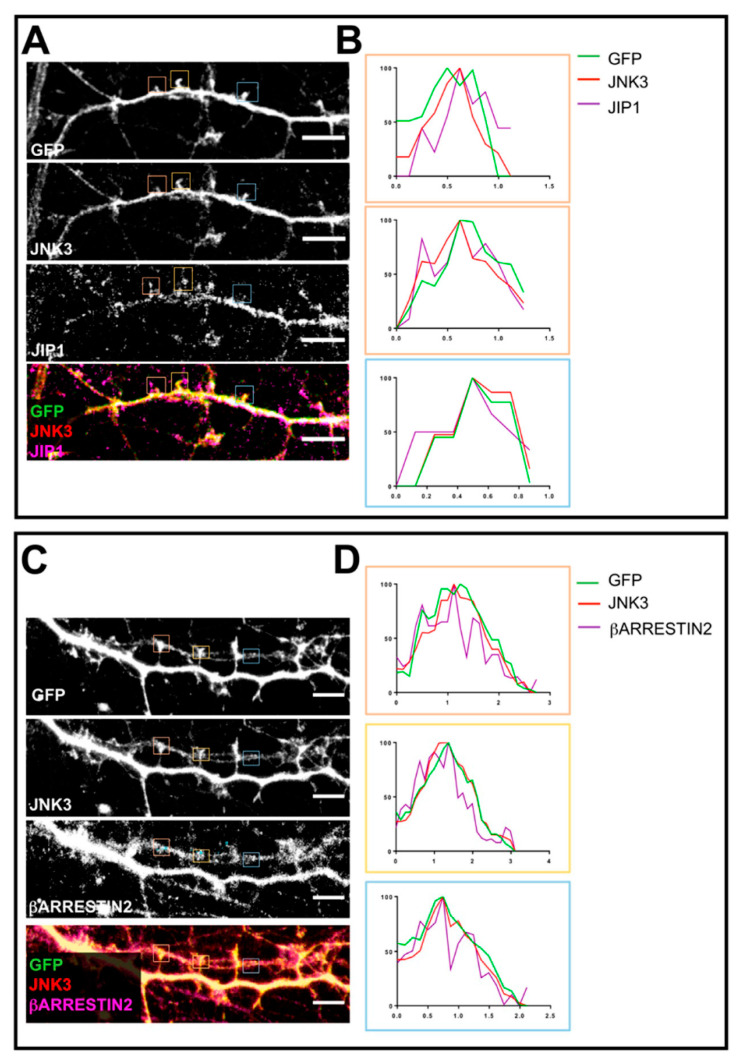
Confocal microscopy outputs used to assess JNK3–JIP1–β-arrestin2–PSD95 colocalization at the dendritic spine level. (**A**) Neurons were fixed and stained with anti-JNK3 (red), anti-JIP1 (violet), and anti-GFP (green). Images were obtained using a Nikon N-SIM confocal microscope and overlaid to assess protein localization. Scale bar of 50 μm. Squares represent the loci analyzed in panel (**B**). (**B**) Intensity profile (green for GFP, red for JNK3, violet for JIP1) representing the values indicated by the squares in panel (**A**). The values are normalized to 100 (arbitrary unit). (**C**) Neurons were fixed and stained with anti-JNK3 (red), anti-β-arrestin2 (violet), and anti-GFP (green). Images were obtained using a Nikon N-SIM confocal microscope and overlaid to assess protein localization. Scale bar of 50 μm. Squares represent the loci analyzed in panel (**B**). (**D**) Intensity profile (green for GFP, red for JNK3, violet for β-arrestin2) representing the values indicated by the squares in panel (**A**). The values are normalized to 100 (arbitrary unit).

To better establish whether the colocalization between JNK3, JIP1, and PSD95 (Figure 1) occurs at the synapse, we used constitutively fluorescent hippocampal neurons derived from Thy1-YFP mice to visualize dendritic spines and determine the presence or absence of JNK3–JIP1 together with PSD95. We co-stained primary hippocampal neurons with JNK3–JIP1 and anti-GFP to better visualize the spines. With confocal microscopy, we found that JNK3 and JIP1, besides being present in the cytoplasm, also showed a dendritic spine localization (Figure 4A). In addition, single-locus profile analysis revealed that JNK3 and JIP1 colocalized (Figure 4B). To assess whether JNK3 also colocalizes with β-arrestin2 at dendritic spines, we used a similar approach to the one described in Figure 4A. We cultured hippocampal neurons derived from Thy1-YFP mice and labeled them with antibodies against JNK3, β-arrestin2, and anti-GFP. Next, we performed confocal microscopy analysis. Immunofluorescence studies showed the postsynaptic localization of both β-arrestin2 and JNK3 (Figure 4C). This was further detailed by profile analysis of single loci of JNK3 and β-arrestin2 (Figure 4D).

### 2.5. Interactions of JNK3, JIP1, PSD95, and β-Arrestin2 in Synaptic Fractions

**Figure 5:**
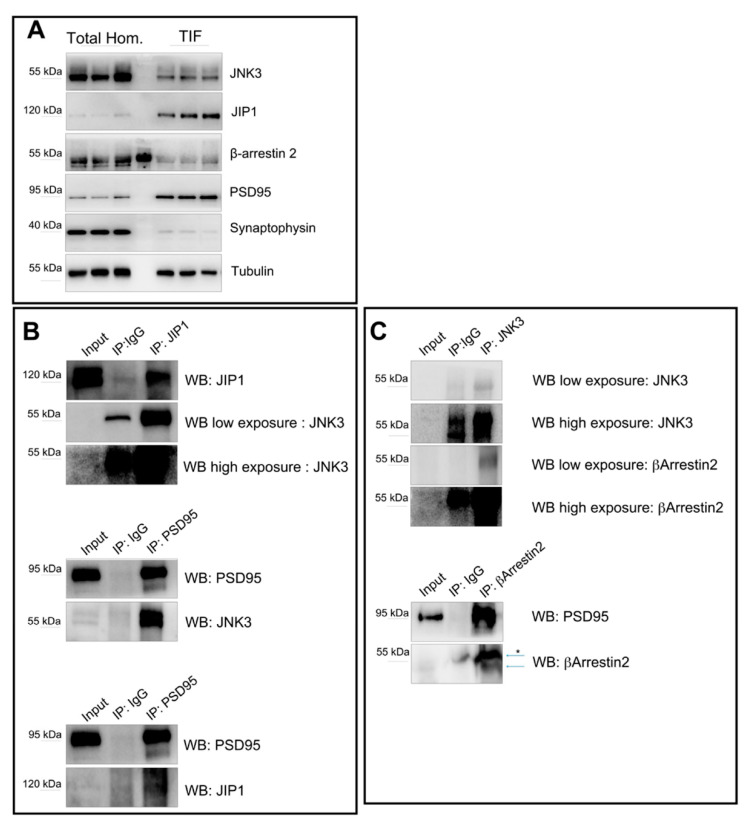
Immunoprecipitation outputs used to assess JNK3–JIP1–β-arrestin2–PSD95 interactions at the synapse. (**A**) Western blots performed on total brain homogenate and Triton-insoluble fraction (TIF) reveal the different amounts of JNK3, JIP1, β-arrestin2, and PSD95 in the two extracts analyzed. Synaptophysin was used as a control for the purity of the TIF preparation. (**B**) JIP1 was immunoprecipitated from the TIF using JIP1 antibody, and immune complexes were analyzed for the presence of JNK3 (rabbit). Immunoprecipitation with IgG antibody was used as a control. PSD95 was immunoprecipitated from the TIF using PSD95-specific antibody, separated by SDS-PAGE, and analyzed with Western blot with anti-PSD95, anti-JNK3 (mouse), and anti-JIP1 antibodies. Immunoprecipitation with IgG antibody was used as a control. (**C**) JNK3 was immunoprecipitated from the TIF using the JNK3 antibody, and immune complexes were analyzed for the presence of β-arrestin2 and JNK3 (rabbit). Immunoprecipitation with IgG antibody was used as a control. β-arrestin2 was immunoprecipitated from the TIF using β-arrestin2-specific antibody, separated by SDS-PAGE, and analyzed by Western blot with anti-PSD95, anti-JNK3, and anti-β-arrestin2 antibodies. Immunoprecipitation with IgG antibody was used as a control. * indicates non-specific bands.

To study whether JNK3, JIP1, β-arrestin2, and PSD95 interact in the spine, we decided to perform immunoprecipitations of JNK3, JIP1, and β-arrestin2 from biochemically purified, postsynaptic protein-enriched fractions (TIF), starting from whole-brain extracts [44]. To this end, we first confirmed that, similar to what we found in the protein extracts obtained from cultured hippocampal neurons, JNK3, JIP1, β-arrestin2, and PSD95 interact in murine whole-brain extracts (Supplemental Figure S1). Next, we confirmed that the proteins are present in the TIF fraction (Figure 5A). We found that JNK3 and β-arrestin2 are likely enriched in the total brain homogenate lysates, compared to the TIF fractions, while JIP1 is increased in the TIF fraction. Of note, in the TIF fraction, the JNK3 antibody recognizes two bands, compared to the one found in the total homogenate (Supplemental Figure S1); this could be due to post-translational modification of the kinase, or the presence of specific isoforms (UniProt P53779-1/2/3). As expected, PSD95 is strongly enriched in the TIF. To further confirm the purity of the biochemical preparations, we used synaptophysin, a vesicle membrane protein enriched in presynaptic terminals [45] as a control. As shown in Figure 5A, synaptophysin is present in the total homogenate, but it is barely detectable in the TIF fraction. Finally, we performed co-immunoprecipitation analysis on TIF fractions. We found that JNK3 interacts with JIP1, β-arrestin2, and PSD95 (Figure 5B,C), thus supporting the confocal evidence of colocalization of the proteins at the synapse.

## 3. Discussion

In this study, we aimed to better characterize the binding between JNK3 and its two scaffold proteins, JIP1 and β-arrestin2, and PSD95. In neurons, the scaffold proteins JIP1 and β-arrestin2 assemble JNK3 signaling modules and regulate different cellular functions. These scaffold proteins can be used to modulate JNK3 functions in separate cellular compartments. However, the biological interaction and localization of JNK3 and its scaffold proteins in the neurons/brain has not been extensively studied. To this end, we used biochemical and microscopy methods to study JNK3 interaction and localization with JIP1 and β-arrestin2, together with PSD95, in cultured hippocampal neurons. We found that JNK3 colocalizes with, and binds to, its scaffolds JIP1 and β-arrestin2. Moreover, it also colocalizes with, and binds to, PSD95, thus suggesting the existence of neuronal protein complexes formed by JIP1–JNK3–PSD95 and β-arrestin2–JNK3–PSD95. These colocalizations and interactions can also be detected in the postsynaptic protein-enriched fraction isolated by TIF fractionation on whole-brain extract, thus suggesting that these complexes may exist at the level of the postsynaptic elements.

**Figure 6:**
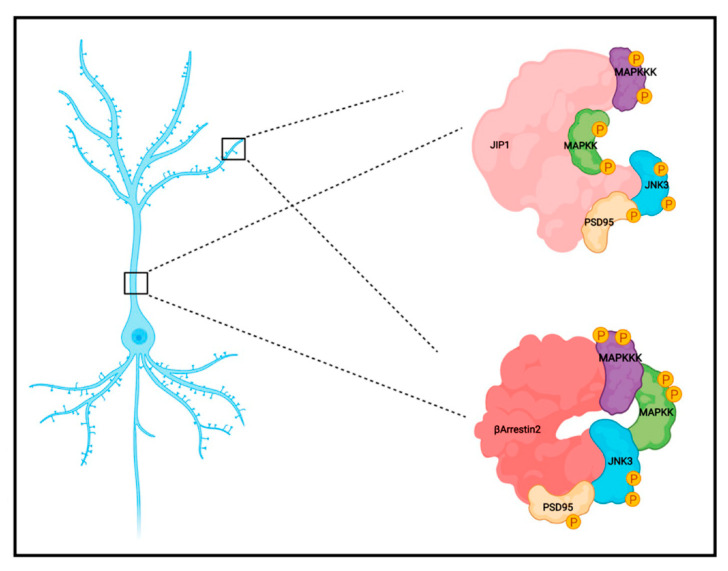
Schematic representation of the protein-protein interactions mediated by JIP1 and b-arrestin2 scaffold proteins. Both scaffold proteins bind upstream MAPK activators, PSD95 and JNK3, thus regulating the signaling cascade in subcellular compartments.

Considering the obtained results, and based on previous studies and models, [26,29,35,46,47,48], we hypothesize an interaction mechanism similar to the one shown in Figure 6, in which scaffold proteins act as cargo to modulate JNK3 signaling in a specific compartment.

Due to the role of JNKs in synaptic dysfunction, the binding of JNK3 to its scaffold proteins in the spine is of utmost interest. In this compartment, the active pool of JNKs strongly modulates AMPA and NMDA receptor trafficking, influencing synaptic strength. JNKs can directly bind to the receptors themselves [49,50], and to PSD scaffolds proteins, such as PSD95 [51], profoundly modifying the dendritic spine morphology and, consequently, function. Importantly, JNK3 can phosphorylate the N-terminal region of PSD-95, and this post-translational modification can potentially affect PSD95 stability and, therefore, the disorganization of the PSD region [52,53,54]. In this context, inhibiting only JNK3, and not all JNKs, in specific subcellular compartments could be a more effective and specific strategy to avoid the potential side effects of pan-JNKs inhibitors.

However, many questions are still unsolved. It will be necessary to study how these interactions change in response to different stress stimuli in the separate cellular compartments, and the contribution of each scaffold protein in the pathological/physiological processes.

Nevertheless, our findings pave the way for the study of new inhibitors of JNK3, which may target its interactions with scaffold proteins for the treatment of synaptopathies. Most JNK drug discovery efforts are focused on developing ATP-competitive inhibitors [55]. However, it is challenging to achieve specificity due to the high homology, in the ATP binding site, among the different JNK proteins [56]. JNK3 protein sequence is 77% identical to JNK2 and 75% to JNK1. The identity of the ATP-binding pocket of the three proteins is instead 98% [57]. For these reasons, most of the JNK3 inhibitors display very weak selectivity, interfering with JNK1/2, and cannot inhibit the action of JNK3 on its targets [58]. On the contrary, strategies targeting the protein-protein interactions that assure the specificity of the JNK3 response have great potential. They could be a promising strategy in drug discovery [59] exhibiting a more specific pharmacological profile [60].

JIP1 and β-arrestin2 proteins organize JNK signaling, and are crucial modulators of JNK3 by providing a binding platform for the kinase and its targets. Targeting specific interacting domains of these scaffold proteins, taking advantage of the substrate competitive mechanism, is a strategy already used to generate one of the most specific JNK inhibitors, D-JNKI1 cell-permeable peptide. This compound, utilizing the crucial JNK-binding domain (JBD) on JIP1, specifically inhibits all JNK isoforms [14,22,26,46]. It has already been tested in different cell types, from pancreatic β-cells [26] to neurons [61], resulting in a potent neuroprotector used against different brain diseases [46,62,63,64,65,66,67,68,69].

Overall, our findings are relevant because 20–33% of drug discovery efforts worldwide are targeting kinases [70] and, since the specificity of these inhibitors is often questioned, defining the molecular mechanisms that may suggest alternative and more specific strategies for targeting kinases (i.e., JNK3) could improve the treatment of complex disorders, such as neurological conditions.

## 4. Materials and Methods

### 4.1. Animals

Procedures involving animals and their care were in accordance with national and international laws and policies (EU Directive 2010/63/EU for animal experiments). The Mario Negri Institute for Pharmacological Research (IRCCS, Milan, Italy) and the Animal Care and Use Committee (IACUC) approved the study, which was conducted according to the institutional guidelines, in compliance with Italian law. The scientific project was approved by the Italian Ministry of Health (cod. 9F5F5.N.WWZ, 2019). Adult mice were euthanized by cervical dislocation [71,72]. Brains were removed and stored at −80 °C until sample processing.

### 4.2. Hippocampal Primary Neuronal Culture

Primary neuronal cultures were obtained from P1-P2 C57BL/6J or Thy1-YFP pups as described in [61], with minor modification. In brief, after brain dissection, hippocampi were incubated with 200 U of papain (P3125, Sigma-Aldrich, St. Louis, MO, USA) (30 min, 34 °C), with trypsin inhibitor (T-9253, Sigma-Aldrich, St. Louis, MO, USA) (45 min, RT), and subsequently mechanically dissociated. Neurons were plated onto ibidi chamber slides (Ibidi GmbH, Germany) (300,000 cells/mL). Plating medium was B27/neurobasal-A (Gibco, Waltham, MA, USA) supplemented with 0.5 mM glutamine (Gibco, Waltham, MA, USA), 100 U/mL penicillin, and 100 μg/mL streptomycin (Gibco, Waltham, MA, USA). We did not treat the cultures with any compound used to eliminate other contaminating cells, such as astrocytes.

### 4.3. Tissue Preparation for Immunoprecipitation Analysis

Half-brain samples were homogenized in ice-cold 1× PBS and 1% Triton X with a complete set of protease inhibitors (Complete; Roche Diagnostics, Basel, Switzerland) and phosphatase inhibitors (Sigma-Aldrich, St. Louis, MO, USA). Samples were centrifuged at 13,000 rpm for 5 min at 4 °C. The supernatants, representing the postsynaptic protein-enriched fraction, were then used for immunoprecipitation analysis.

### 4.4. Triton-Insoluble Fractionation (TIF)

Subcellular fractionation was as reported by [44], with minor modification. Briefly, half-brain samples were homogenized with a glass/Teflon Potter apparatus in 0.32 M ice-cold sucrose buffer containing the following concentrations (in mM): 1.0 HEPES, 1.0 MgCl2, 1.0 EDTA, 1.0 NaHCO3, and 0.1 PMSF, at pH 7.4, with a complete set of protease inhibitors (Complete; Roche Diagnostics, Basel, Switzerland) and phosphatase inhibitors (Sigma-Aldrich, St. Louis, MO, USA). Samples were centrifuged at 1000× *g* for 10 min. The supernatant (S1) was then centrifuged at 3000× *g* for 15 min to obtain a crude membrane fraction (P2 fraction). The pellet was dissolved in buffer containing 75 mM KCl and 1% Triton X-100, as well as protease and phosphatase inhibitors, and centrifuged at 100,000× *g* for 1 h. The supernatant was stored and referred to as TSF (Triton-soluble fraction, S4). The final pellet (P4), referred to as TIF, was homogenized in a glass/glass Potter apparatus in PBS and 0.5% Triton X-100, with a complete set of protease and phosphatase inhibitors, and stored at −80 °C until immunoprecipitation analysis.

### 4.5. Immunoprecipitation

Cell extracts, total brain homogenates, and TIF extracts (300 μg) were incubated overnight at 4 °C with 1 μg of anti-JNK3, anti-PSD95, anti-β-arrestin2, anti-JIP1, or anti-HA antibodies. Dyna beads (Thermo Fisher Scientific, Waltham, MA, USA) or protein A/G agarose (GeneSpin, Milano, Italy) were then incubated with the lysate for 1 h at 4 °C. The beads were washed five times with PBS1x and Triton 0.5%, and bound proteins were eluted with the elution buffer and analyzed by Western blotting. At least three independent experiments were performed, and representative images are shown.

### 4.6. Western Blotting

Immunoprecipitated proteins were separated by 10% SDS polyacrylamide gel electrophoresis. PVDF membranes were blocked in Tris-buffered saline (5% no-fat milk powder, 0.1% Tween 20), (1 h, RT). Primary antibodies (anti-JNK3, Invitrogen, Thermo Fisher Scientific, Waltham, MA, USA, #PA5-14421, mAB #2305; anti-PSD95 Clone 6G6 Cayman Chemical, Michigan, USA; anti-JIP1 AbCam Cambridge, UK Ab24449; and anti-β-arrestin2, Thermo Fisher Scientific, Waltham, MA, USA, # PA1-732) were diluted in BSA 5%, 0.1% Tween 20 and incubated overnight at 4 °C. Blots were developed using horseradish peroxidase-conjugated secondary antibodies (Santa Cruz Biotechnology, Dallas, TX, USA) and the ECL chemiluminescence system (Bio-Rad, Hercules, CA, USA).

### 4.7. Immunofluorescence

Immunofluorescence experiments were performed according to [73]. Briefly, hippocampal primary cultures at DIV14 (see above) were fixed in 4% paraformaldehyde (PFA) and 4% sucrose solution for 30 min, followed by permeabilization with phosphate-buffered saline (PBS) at pH 7.4, containing 0.5% Triton X-100, for 3 min. Co-cultures were first blocked for 1 h in PBS containing 1% BSA, 0.2% Triton X-100, and subsequently incubated overnight at 4 °C with primary antibodies in PBS containing 1% BSA and 0.2% Triton X-100. The following antibodies were used: anti-GFP (AbCam Cambridge, UK Ab290), anti-PSD95 (AbCam Cambridge, UK Ab12093), anti-JIP1 (AbCam Cambridge, UK Ab24449), anti-β-arrestin2 (AbCam Cambridge, UK Ab31294), anti-JNK3 (Invitrogen, Thermo Fisher Scientific, Waltham, MA, USA #PA5-14421). Cells were finally incubated with secondary antibodies (AlexFluor Antibody, Thermo Fisher Scientific, Waltham, MA, USA) for 1 h at room temperature. A concentration of 2 mg/mL Hoechst (Thermo Fisher Scientific, Waltham, MA, USA, 33342) was used to stain nuclei. ProLong Glass Antifade Mountant (Thermo Fisher Scientific, Waltham, MA, USA) was used as a mounting agent.

### 4.8. Confocal Microscopy and Structured Illumination Microscopy (SIM)

Confocal microscopy and SIM were performed according to [74]. In brief, images were acquired using Nikon A1 confocal microscope and Nikon N-SIM microscope. Confocal images were collected using a 100× objective with a stack thickness of between 0.24 and 0.48 μm. SIM images were collected in 3D-SIM mode. After their acquisition, Fiji (ImageJ-1.8.0_172) software was used to process the raw images.

## Data Availability

Data are available upon request to the corresponding author.

## Supplementary Materials

The following supporting information can be downloaded at: https://www.mdpi.com/article/10.3390/ijms23084113/s1.
