# Supplementary material for: Colocalization and Interaction Study of Neuronal JNK3, JIP1, and β-Arrestin2 Together with PSD95
Source: Int J Mol Sci. 2022 Apr 8;23(8):4113. doi: 10.3390/ijms23084113 (PMC9024448; doi:10.3390/ijms23084113)
Supplement: Supplementary file 1 [file ijms-23-04113-s001.zip › ijms-1451577-Supplementary.pdf]

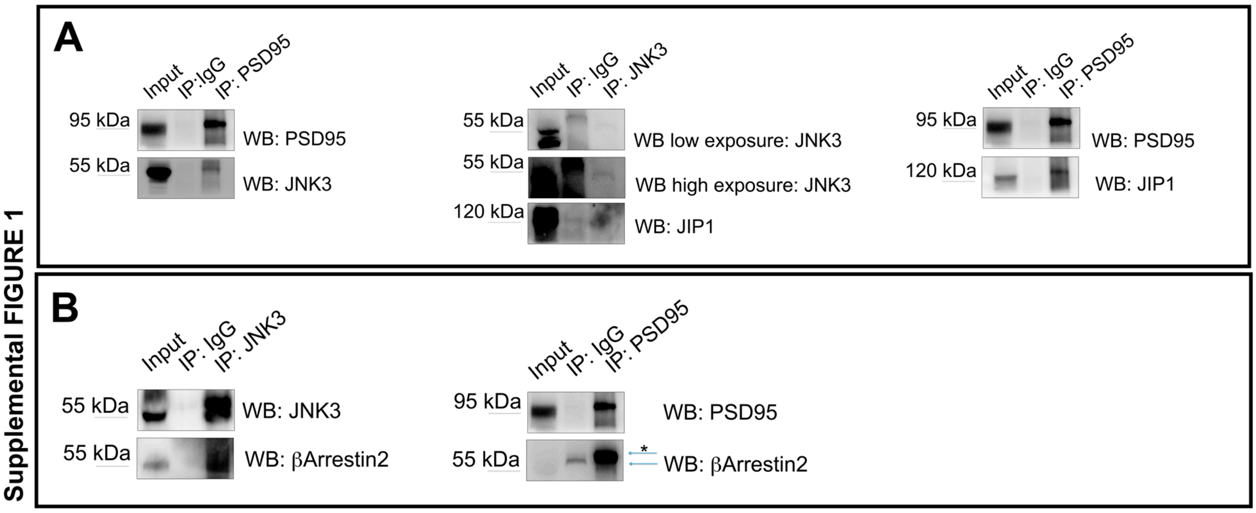


**Supplementary Figure S1. Immunoprecipitations to assess JNK3-JIP1/ß-arrestin2-PSD95 colocalization in the whole brain homogenate.** (A) PSD95 was immunoprecipitated from the whole mice brain homogenate using PSD95-specific antibody, separated by SDS-PAGE and analyzed with Western Blot with anti-PSD95, anti-JNK3 (Rabbit), and anti-JIP1 antibody. Immunoprecipitation with IgG antibody was used as a control. JNK3 was immunoprecipitated using JNK3 antibody, and immune complexes were analyzed for the presence of JIP1 and JNK3 (Mouse). Immunoprecipitation with IgG antibody was used as a control. (B) PSD95 was immunoprecipitated from whole mice brain homogenate using PSD95-specific antibody, separated by SDS-PAGE and analyzed with Western Blot with anti-PSD95 and anti-ß-arrestin2 antibody. Immunoprecipitation with IgG antibody was used as a control. JNK3 was immunoprecipitated using JNK3 antibody, and immune complexes were analyzed for the presence of ß-arrestin2 and JNK3 (rabbit). Immunoprecipitation with IgG antibody was used as a control. * Indicates non-specific bands.
